# Does the resumption of international tourism heighten COVID-19 transmission?

**DOI:** 10.1371/journal.pone.0295249

**Published:** 2024-02-07

**Authors:** Paravee Maneejuk, Panuwat Sukinta, Jiraphat Chinkarn, Woraphon Yamaka

**Affiliations:** Center of Excellence in Econometrics, Chiang Mai University, Chiang Mai, Thailand; University of City Island, CYPRUS

## Abstract

Reopening countries also carries the risk of another wave of infections in many parts of the world, raising the question of whether we are ready to reopen our countries. This study examines the impact of reopening countries to receive foreign tourists on the spread of COVID-19 in 2022, encompassing 83 countries worldwide. We employ spatial quantile models capable of analyzing the spatial impact of tourism on the spread of the virus at different quantile levels. The research categorizes countries into three groups: low infection rate (10th-30th quantiles), moderate infection rate (40th-60th quantiles), and high infection rate (70th-90th quantiles). This allows for a more comprehensive and detailed comparison of the impacts. Additionally, considering the spatial dimension enables the explanation of both the direct and indirect effects of tourists on the country itself and neighboring countries. The findings reveal that the number of international tourists has a significant effect on the COVID-19 infection rate, particularly in countries with high initial infection rates. However, countries that effectively controlled their infection rates at a low level could maintain a low infection rate even after reopening to foreign tourists. It is also observed that reopening a country’s borders negatively impacts the infection rate of neighboring countries. These important findings imply that governments of highly infected countries should shift their focus towards bolstering their economy by promoting domestic tourism and should delay reopening until the number of infections decreases.

## 1. Introduction

The COVID-19 pandemic has had far-reaching consequences on global economies, with the tourism industry being one of the hardest-hit sectors [1]. Travel restrictions and border closures imposed worldwide have dealt a severe blow to countries heavily reliant on tourism, resulting in enduring and inevitable economic consequences. Previous studies have extensively documented the empirical evidence highlighting the economic and tourism-related ramifications of COVID-19. However, the dynamic nature of the current pandemic situation, as highlighted by Ghebreyesus [2], has compelled countries to consider reopening their borders and welcoming an influx of tourists. While reopening borders is crucial for economic revitalization, it also introduces the risk of heightened local transmission within destination countries. This study aims to address a previously overlooked aspect: whether reopening borders to accommodate tourists will contribute to an increased rate of domestic transmission. Moreover, it seeks to investigate whether countries with varying levels of domestic infection rates will experience differing impacts from international tourists. The findings of this study will offer valuable policy recommendations to strike a balance between stimulating the economy and mitigating the risks associated with domestic transmission of the pandemic.

Since the emergence of the novel coronavirus in December 2019 in Wuhan, China, its rapid global spread has been facilitated by human mobility and travel between regions. Faridi et al. [3] conducted a study that revealed humans as the fastest carriers of the infection, facilitating its transmission from person to person. Consequently, when individuals travel from one place to another, they increase the likelihood of spreading the disease to different locations more easily. This phenomenon is exemplified by research conducted by Brownstein, Wolfe, and Mandl [4], who confirmed that air travel between countries was a significant factor in importing influenza cases into the United States. Moreover, domestic air travel also contributed to the dissemination of influenza between regions within the country. Conversely, human mobility can also lead to the introduction of infectious diseases from other locations. Findlater and Bogoch [5] observed that the spread of infectious diseases across various countries often stemmed from human air travel. The rapid connectivity provided by air travel between destination and origin points facilitated the swift and extensive dissemination of new outbreaks. Consequently, during the initial stages of the COVID-19 pandemic, when travel restrictions and policies were not yet in place, the virus spread rapidly worldwide due to unrestricted human movement.

The act of traveling for tourism is an essential human activity that necessitates mobility, but it also carries the potential to facilitate the transmission of infectious diseases to destination areas. The convenience and speed of modern travel contribute to this phenomenon. Nunkoo et al. [6] emphasized that domestic tourism acts as a vector for the rapid spread of viruses during the initial stages of an outbreak. Additionally, Tantrakarnapa et al. [7] focused on the factors influencing the spread of COVID-19 in Thailand and identified a significant correlation between the number of tourists, both domestic and international, and the number of COVID-19 infections and cases in the country. Consequently, it can be argued that tourism-related travel is one of the factors responsible for the swift and extensive outbreak of new infectious diseases.

As a result of the aforementioned factors, many countries worldwide have implemented various measures to mitigate the spread of diseases, including border closures, Vaccine passports, travel restrictions for tourism, and internal lockdown measures [2, 8]. These efforts have proven effective in reducing the number of COVID-19 cases and slowing down the epidemic. However, these measures have also had significant economic implications, particularly for countries heavily reliant on tourism, leading to an economic slowdown [9]. Therefore, in order to maintain overall economic stability and support the tourism sector during disease outbreaks, it becomes crucial to implement appropriate tourism policies. One key aspect of these policies involves making informed decisions regarding the reopening of borders and welcoming tourists only when the country is adequately prepared.

**Fig 1:**
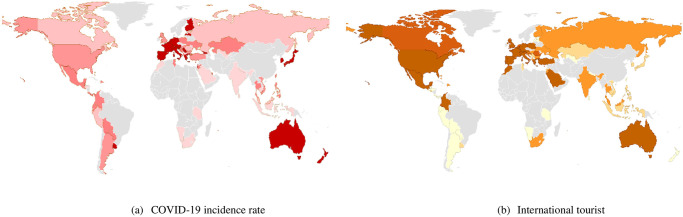
(1) COVID-19 infection rate per capita and (2) the number of international tourists visiting each country between January and December 2022. Note: Darker areas represent higher values, lighter areas represent lower values, and grey areas mean no data. This plot is done by the ggplot r package. Source: Author’s plot.

To provide a more visual representation, this study presents a graph showing the cumulative number of international tourists who began visiting after the COVID-19 pandemic or during 2022, as well as the cumulative number of COVID-19 infections during the same period for 83 countries, as shown in Fig 1. It is observed that the number of international tourists and the number of infections in each country have a relatively similar pattern. In other words, countries that have a higher number of international tourists also tend to have a higher number of infections within the country. This can be observed from the relatively darker shade that corresponds to both the left and right sides of Fig 1. Furthermore, it has been observed that countries with contiguous territories often exhibit similar patterns of infection, or they form distinct clusters. For example, in European countries, a clear grouping of similar infection patterns can be observed. This reflects that geographical factors may contribute to similar impacts in each region or that neighboring countries may experience similar effects. This is consistent with the study by Ahmed and May [10] that examined the spread of COVID-19 from one country to neighboring countries. It found that the spread of COVID-19 from one country significantly affected the number of infected cases in the neighboring areas. This supports the notion that geographical factors, such as contiguous or proximate countries and short-distance transportation routes, may contribute to an increased tendency for disease transmission. Consequently, it can lead to the escalated spread of the disease in adjacent areas, resulting in the formation of distinct clusters.

The role of tourist travels in facilitating the spread of diseases to different regions cannot be underestimated. Ahmed and May [10] conducted a study examining the spread of COVID-19 to neighboring countries and revealed that a high prevalence within one country could significantly impact the neighboring areas. However, limited research exists on the specific relationship between travel for tourism purposes and disease transmission. Tantrakarnapa, Bhopdhornangkul, and Nakhaapakorn [7] conducted a recent study in Thailand, which found a correlation between the number of tourists (both domestic and international) and the number of confirmed COVID-19 cases and patients. Another study by Anzai and Nishiura [11] suggested that increased domestic tourism may contribute to a rise in COVID-19 infections. However, these findings are primarily based on country-level analysis and do not fully consider spatial effects in the relationship between COVID-19 spread and tourism. Therefore, the objective of this study is to explore the geographical dimensions of disease transmission through tourism-related travel. While existing studies often focus on analyzing the impact of COVID-19 on tourism, it is crucial to recognize that tourism can also contribute to the spread of the disease. Hence, this study aims to investigate the impact of international tourism on the infection rate, particularly during the period of reopening international arrivals in numerous countries in 2022. By examining the spatial effects and considering the interplay between tourism and disease transmission, this study seeks to provide valuable insights into the specific relationship between tourism-related travel and the spread of COVID-19.

The determination of the spread of COVID-19 in a country is not solely driven by domestic factors but is also influenced by variables present in neighboring countries, especially those in close proximity [12]. Moreover, the spatial impact of other locations tends to decrease with distance, with the effect of a distant country considered less significant than that of a nearby one. As a result, spatial dependence can weaken or introduce bias to the estimators derived from the ordinary least squares (OLS) method [13]. To address these concerns, a spatial regression model is utilized in this study. However, the spatial regression model still shares certain limitations with traditional regression models. Firstly, it assumes a strict normal distribution, which is often difficult to meet with actual spatial observations [14]. Secondly, the spatial regression model merely establishes a homogeneity relationship between dependent and independent variables, whereas spatial entities are often heterogeneous. Regions with varying degrees of COVID-19 spread may respond differently to tourism. In this regard, quantile regression models provide a promising approach to handling outliers and capturing more detailed information from the data.

The primary contribution of this study lies in its investigation of the influence of human mobility for tourism on the rate of COVID-19 infections. By categorizing countries into different infection rate groups based on quantiles, this study provides valuable insights for countries considering the reopening of their borders to tourists. Policymakers and public health authorities can benefit from these insights to make informed decisions and implement measures that mitigate the risk of virus transmission while supporting tourism. Furthermore, this study acknowledges the potential spatial effects that could impact neighboring countries, enhancing the understanding of cross-border transmission dynamics. The utilization of spatial quantile regression models as a robust analytical tool allows for an in-depth analysis of the data, enabling the identification of spatially varying effects on the relationship between tourism and the spread of the pandemic. By analyzing cumulative data on COVID-19 infections and tourist arrivals across 83 countries from January to December 2022, this study contributes to the existing knowledge on the role of tourism in shaping the patterns of COVID-19 transmission. Researchers and scholars interested in the intersection of tourism and public health can benefit from this comprehensive dataset and analytical approach to further explore this important area of study.

The paper is organized into the following sections: a literature review in Section 2, data and spatial quantile regression models in Section 3, results in Section 4, Section 5 engages in discussion, Section 6 presents the limitations and recommendations for future research, and Section 6 concludes with recommendations.

## 2. Literature review

### 2.1 Human mobility and the spread of diseases

The resumption of international tourism amid the ongoing COVID-19 pandemic has significant implications for disease transmission, which can be understood through epidemiological theory. Epidemiology, as a discipline, provides valuable insights into disease spread dynamics. Key factors include human-to-human transmission, the reproduction number (R0), asymptomatic and presymptomatic transmission, and the occurrence of superspreader events [15]. In line with the observations made by Lim [16] who advocated that globalization in trade and population mobility are significant drivers of the emergence, re-emergence, and transmission of infectious diseases in the modern era, this study delves into the role of international tourism as a key component of population mobility. This perspective aligns with the findings of Iyaniwura et. al. [17], who have reaffirmed the central role of human mobility in the ongoing spread of COVID-19. Against the backdrop of rapid globalization in trade and population mobility in the twenty-first century, densely populated urban centers in highly globalized countries have evolved into focal points for international tourism. These urban centers are intricately connected to the global economy, attracting travelers from diverse regions. As a result, the impact of international tourism on the transmission of COVID-19 may exhibit variations across countries, contingent upon their degree of globalization, as noted by Bickley et al. [18].

Extensive research conducted in the past has consistently demonstrated the pivotal role of human mobility in the dissemination of communicable diseases, providing valuable insights into the mechanisms through which diseases can rapidly propagate across regions and even continents. The findings from these studies highlighted the profound impact of increased human mobility, particularly through trade, migration, and tourism, on the global spread of infectious diseases. Notably, studies by Findlater and Bogoch [5] have shed light on the transmission dynamics of diseases such as Zika virus and MERS-CoV across different regions. Their research highlighted how increased human mobility, facilitated by trade, migration, and tourism, has contributed to the swift dissemination of these diseases. Similarly, investigations by Brownstein, Wolfe, and Mandl [4] have elucidated the association between international air travel and the introduction of influenza cases into the United States, with domestic air travel further exacerbating its spread within the country. These findings emphasize the significant role played by air travel in enabling the movement of infected individuals and the subsequent transmission of infectious diseases. Furthermore, Fang et al. [19] worked on the transmission of SARS in China and identified major highways and air travel as influential factors in the rapid dissemination of the disease to neighboring provinces. Their research demonstrated how the interconnectedness facilitated by transportation routes, particularly air travel, has acted as a catalyst for the swift and extensive transmission of emerging infectious diseases. These studies collectively underscore the global health threat posed by the interconnected nature of our modern world, which amplifies the potential for the rapid and widespread transmission of infectious diseases.

### 2.2 Tourism on the spread of COVID-19

Extensive research has consistently highlighted the significant impact of human mobility, particularly in the context of tourism, on the spread of COVID-19. Numerous studies have specifically demonstrated the influential role of air travel in the rapid global dissemination of the virus [20, 21].). The sheer volume of tourists, both domestic and international, has been found to be strongly correlated with the number of COVID-19 cases, as observed in countries like Thailand [7]).

Popular tourist destinations and transportation hubs, including airports, have been identified as significant sources of COVID-19 transmission within countries. The concentration of people in these areas, along with the movement of individuals from different regions, greatly amplifies the risk of viral spread. Moreover, government-led domestic tourism campaigns aimed at revitalizing local economies have inadvertently contributed to a surge in COVID-19 cases associated with travel [11, 22]. For instance, Japan’s "Go To Travel" campaign has been linked to an increase in COVID-19 infections [23].. Similarly, initiatives promoting activities like dining out have accelerated the transmission of COVID-19 to neighboring regions [24]. Tamura, Suzuki, and Yamaguchi [22] quantified the impact of the Japanese government’s tourism campaign and found that it raised the rate of cases by 23.7% to 34.4% between July 30 and August 4, 2020. While these endeavors are intended to revive industries and stimulate economic recovery, they have sometimes disregarded the potential risks associated with increased human mobility and social interactions. It is crucial to acknowledge that disease prevalence within a domestic context has implications that extend beyond national borders. Regions with high levels of COVID-19 transmission can significantly affect neighboring areas, underscoring the necessity for international cooperation in mitigating the virus’s spread [10]. Governments have implemented various measures such as border closures, quarantines, travel restrictions, and lockdowns to limit human mobility and reduce COVID-19 transmission. By recognizing the interconnected nature of the pandemic, these measures are crucial for safeguarding public health and facilitating a coordinated global response.

Research has shown that border closures have proven effective in reducing cases and delaying epidemics between regions. However, the effectiveness of travel limitations on domestic tourism diminishes over time [6, 25]). Local factors, including community transmission rates, healthcare capacity, and adherence to preventive measures, should be carefully considered in understanding disease transmission patterns during different stages of outbreaks [26]).

### 2.3 Factors influencing the spread of diseases

Previous research has yielded substantial evidence demonstrating the significant role of tourism activities and human mobility in the dissemination of various infectious diseases, including the ongoing COVID-19 pandemic. Studies have consistently highlighted the influential contribution of travel-related factors, such as major transportation routes that interconnect regions and the extensive network of air travel, in facilitating the transmission of diseases [5, 27]). Additionally, a growing body of literature has identified several additional determinants that exert an influence on disease spread. These include population density within urban areas, the concentration of older adults within communities, the intensity of public transportation utilization, and various economic parameters encompassing employment rates, retail sales per area, and industrial value, among others. Furthermore, fundamental economic indicators like Gross Domestic Product (GDP) per capita and Gross National Income (GNI) have been consistently associated with the propagation of infectious diseases, with empirical investigations revealing a positive correlation between these indicators and infection rates as well as mortality rates attributed to communicable diseases [28–30]. The convergence of these research findings reveals the intricate interplay among travel patterns, population density dynamics, and socioeconomic circumstances, shaping the intricate landscape of infectious disease transmission.

Another interesting factor is the vulnerability of the population and the number of unskilled laborers, which have been found to be positively associated with the spread of COVID-19. These individuals often face challenges in accessing healthcare services, including limited availability of vaccines, and may find it difficult to adhere to lockdown measures due to their dependence on daily labor for sustenance. As a result, these factors increase the risk of COVID-19 transmission within communities [31]. Conversely, factors related to robust public health infrastructure, such as hospital density, healthcare personnel, hospital capacity, and availability of medical resources, have consistently shown a negative association with infection rates. These factors contribute to effective disease control and mitigation efforts [32].

## 3. Data and methodology

### 3.1 Data

The cross-sectional dataset used in this study comprises information from 83 countries worldwide in the year 2022. Specifically, this dataset encompasses data collected from January to December, with our analysis focusing on either the summation of data or the most recent data available at the end of the year. The selected time period is particularly relevant as it coincides with the reopening efforts of many countries following the wave of the pandemic. The sample of countries was chosen to ensure representation from different continents, including 24 countries from Asia, 29 from Europe, 2 from Oceania, and 20 from North and South America, as well as 8 countries from Africa. This diverse selection aimed to capture a range of regional variations and facilitate a comprehensive analysis of the relationship between tourism and disease spread.

In this study, the focus centers on analyzing factors that influence the COVID-19 infection rate within countries. The dependent variable, "COVID-19 infection rate per population" (CASE), serves as a measure of the spread of the disease. The main independent variable of interest is the number of tourists entering each country (ARRIVAL), which is hypothesized to explain variations in the infection rate. To account for potential confounding factors, the analysis encompasses several control variables. These encompass the country’s economic level (GDP), population density (POP), a stringency index that gauges the effectiveness of epidemic control measures (STR), the rate of full vaccination coverage (FULLVAC), the proportion of the elderly population (AGE65), and trade openness (TRADE).

Theoretical explanations regarding the influence of various indicators on the spread of COVID-19 are both intricate and crucial. A higher Gross Domestic Product (GDP) typically signifies greater economic resources, leading to improvements in healthcare infrastructure, medical facilities, and treatment accessibility. Consequently, this aids in reducing the virus’s transmission [29, 30]. The stringency index, which reflects the imposition of strict epidemic control measures such as lockdowns, mask mandates, and travel restrictions, plays a pivotal role in mitigating virus transmission by reducing opportunities for contagion and preventing super-spreader events [29]. In terms of population density, it has been observed to increase coronavirus transmission in various contexts. Given that face-to-face contact is a primary mode of virus transmission, areas with higher population densities tend to exhibit higher infection rates. A preliminary literature review has shown that density is a statistically significant and positive predictor of COVID-19 [28]. Furthermore, achieving a higher rate of full vaccination coverage is instrumental in attaining herd immunity, effectively dampening the virus’s spread, reducing the severity of outbreaks, and alleviating the strain on healthcare systems. The proportion of the elderly population significantly influences the virus’s impact since the elderly are more susceptible to severe illness and fatalities [33]. Regions with a larger elderly demographic face heightened risks, emphasizing the necessity of stringent protective measures [34].

Additionally, trade openness can impact the spread of COVID-19 due to global interconnectedness. While it may facilitate the importation of the virus, it can also expedite the procurement of essential medical supplies, vaccines, and information sharing. Trade openness can also influence child health through government spending on healthcare, fueled by tax revenues generated from trade activities [35]. This suggests that increased trade activities in the economy lead to higher government revenues, subsequently increasing healthcare spending on child health. These variables emphasize their significance as predictors of COVID-19 spread. By incorporating these control variables, our objective is to mitigate potential biases and gain a more comprehensive understanding of the relationship between tourism and COVID-19 transmission.

Table 1 presents the descriptive statistics of the data used in this study, and the lsit of sample countries is laid out Table A1 in S1 Appendix.

**Table 1:** Basic statistics and descriptions of variables.

Variable	Max	Min	Mean	Skewness	SD	Description (Unit)	Source
**CASE**	0.55	0.00	0.13	1.07	0.14	Rate of COVID-19 infection per population (%)	OUR WORLD DATA
**POP**	20,546.77	3.07	626.43	6.81	2501.40	Population density per square kilometer (people)	THE WORLD BANK
**GDP**	116,935.62	2442.80	27,109.83	1.49	22,182.46	Gross Domestic Product per capita (US dollars per person per year)	THE WORLD BANK
**ARRIVAL**	71.51	0.07	3.79	2.52	5.56	Cumulative number of tourist arrivals in 2022 (million people)	CEIC DATA
**STR**	61.24	11.30	30.38	0.65	10.10	Stringency index (epidemic control index)	OUR WORLD DATA
**FULLVAC**	105.75	20.49	69.85	-0.73	15.08	Percentage of the population is considered fully vaccinated (%)	OUR WORLD DATA
**AGE65**	27.05	1.31	11.98	0.16	6.30	Rate of the population aged 65 and above (%)	OUR WORLD DATA
**TRADE**	402.51	25.	101.40	2.44	72.06	Traded openness (the sum of imports and exports normalized by GDP) (%)	THE WORLD BANK

### 3.2 Methodology

To account for the spatial impact of tourism on the spread of the pandemic, this study employs the spatial quantile Regression model. This model allows for the analysis of spatial effects at different quantile levels. By considering different quantiles, we can obtain distinct coefficients for factors, thus offering a more comprehensive understanding of the relationship between factors and the spread of COVID-19. Building upon the work of Kostov [36], this study combines a spatial model with quantile regression, as proposed by Koenker and Hallock [37], to investigate the influence of tourism on the spread of COVID-19. This approach does not impose assumptions on data distribution, allows for the heterogeneity of influential characteristics on the dependent variable, and accounts for spatial dependence [38]. To our knowledge, the application of the spatial quantile model to this topic remains unexplored in existing literature.

Three specifications of the spatial quantile regression model are utilized: the spatial quantile lag model (SQLM), the spatial quantile error model (SQEM), and the spatial quantile durbin model (SQDM). Each model offers unique insights into the spatial relationship between tourism and the spread of COVID-19. Furthermore, to validate the presence of spatial correlation in the variables of interest, the researcher conducts a test using the Moran I statistic. This statistical test provides an indication of whether spatial autocorrelation exists among the variables, which suggests the presence of spatial dependence. By examining the Moran I statistic, we confirm the presence of spatial correlation and ensure that the spatial nature of the data is appropriately accounted for in the analysis.

### 3.3 Spatial autocorrelation

When evaluating the spatial correlation of each variable considered in this study, the analysis focuses on assessing the extent to which each variable exhibits spatial influence within its own geographical area and neighboring regions. To conduct this examination, the Moran I test statistic is employed. This statistical test allows for the assessment of spatial autocorrelation and helps determine the presence of spatial dependence among the variables under investigation. The Moran I test statistic can be written as

$$I=\frac{n\sum_{i=1}^{n}\sum_{j=1}^{n}w_{ij}(x_{i}-\bar{x})(x_{j}-\bar{x})}{\sum_{i=1}^{n}{\left(x_{i}-\bar{x}\right)}^{2}\sum_{i=1}^{n}\sum_{j=1}^{n}w_{ij}}$$ (1)

where *x*_*i*_ and *x*_*j*_ represent the variables of country *i* and country *j*, respectively. $\bar{x}$ represents the average of the studied variables (both independent and dependent variables). *n* represents the number of countries. The spatial weight, denoted as *w*_*ij*_, measures the spatial influence between countries *i* and *j*. We assign a value of spatial weight as 1 if country *i* and country *j* are adjacent and 0 if country *i* and country *j* are not adjacent. In this study, the interpretation of Moran’s I statistic is as follows: If the value of Moran’s I is greater than 0, it indicates that the variable exhibits positive spatial autocorrelation, meaning it has a similar pattern within its own area and neighboring areas. Conversely, if Moran’s I is less than 0, it suggests negative spatial autocorrelation, indicating that the variable has a dissimilar pattern within its own area and neighboring areas. When Moran’s I is equal to 0, it implies no spatial correlation, indicating the absence of a spatial pattern or spatial dependence.

### 3.4 Spatial quantile regression models

The three aforementioned models can be explained as follows:

1) Spatial quantile lag model (SQLM)

The model considers the spatial effect in the dependent variable, which in this study is denoted as CASE_*i*_. This model takes into account the spatial dependence and examines how the spatial lag of the dependent variable influences the quantiles of the response variable. By incorporating the spatial lag term, the model captures the influence of neighboring areas on the spread of the pandemic, providing insights into the spatial dynamics of the disease transmission. The model can be written as follows:

$$\begin{array}{l} {\text{lnCASE}}_{i}=\rho (\tau )w_{ij}{\text{lnCASE}}_{i}\text{+}\beta_{1}\text{(}\tau {\text{)lnARRIVAL}}_{i}+\beta_{2}\text{(}\tau {\text{)lnAGE65}}_{i}+\beta_{3}\text{(}\tau {\text{)lnSTR}}_{i}+\beta_{4}\text{(}\tau {\text{)lnFULLVAC}}_{i}\\ \;\;\;\;\;\;\;\;\;\;\;\;\;\;\;\;\;\;\;\;+\beta_{5}\text{(}\tau {\text{)lnGDP}}_{i}+\beta_{6}\text{(}\tau {\text{)lnPOP}}_{i}+{\text{lnTRADE}}_{i}+\varepsilon_{i},\;\;\;\;\varepsilon_{i}\sim N(0,\sigma^{2}\text{(}\tau \text{)}), \end{array}$$ (2)

where lnCASE_*i*_ represents the value of the dependent variable for country *i*, *i* = 1, …, *n*, the independent variables consist of lnARRIVAL_*i*_, lnAGE65_*i*_, lnSTR_*i*_, lnFULLVAC_*i*_, lnGDP_*i*_, lnPOP_*i*_ and lnTRADE_*i*_ for country *i*. In the above model, we can observe the inclusion of the spatial influence term in the varible CASE_*i*_ denoted as *w*_*ij*_CASE_*i*_ and it corresponding spatial lag parameter, denoted as *ρ*(*τ*). If the coefficient of *ρ*(*τ*) at a certain quantile level is not equal to zero, it indicates that spatial influence is affecting the variable due to spatial spread. For the error term *ε*_*i*_, it follows a normal distribution with a mean of zero and a variance of *σ*^2^(*τ*). Note that *τ* is the corresponding quantile of the model, which ranges from 0 to 1.

2) Spatial quantile error model (SEQM)

This model is proposed to analyze the potential spatial impact that may exist in the error term, even when the spatial influence is not present in the explanatory and independent variables. In traditional regression models, the assumption is that the error terms are independently and identically distributed. However, in the presence of spatial autocorrelation, this assumption may be violated, leading to biased and inefficient parameter estimates. The SEQM accounts for spatial autocorrelation by introducing a spatial error term into the quantile regression framework. This spatial error term captures the spatial dependence in the model residuals, allowing for the estimation of robust quantile regression coefficients. By incorporating the spatial error term, the SEQM enables the identification of spatially varying effects on the quantiles of the response variable, providing valuable insights into the spatial patterns of the relationship between tourism and the spread of the pandemic. The model can be written as follows:

$$\begin{array}{l} {\text{lnCASE}}_{i}=\beta_{1}\text{(}\tau {\text{)lnARRIVAL}}_{i}+\beta_{2}\text{(}\tau {\text{)lnAGE65}}_{i}+\beta_{3}\text{(}\tau {\text{)lnSTR}}_{i}+\beta_{4}\text{(}\tau {\text{)lnFULLVAC}}_{i}\\ \;\;\;\;\;\;\;\;\;\;\;\;\;\;\;\;\;\;\;\;+\beta_{5}\text{(}\tau {\text{)lnGDP}}_{i}+\beta_{6}\text{(}\tau {\text{)lnPOP}}_{i}+{\text{lnTRADE}}_{i}+\lambda (\tau )w_{ij}{\text{u}}_{i}+\varepsilon_{i},\;\;\;\;\varepsilon_{i}\sim N(0,\sigma^{2}\text{(}\tau \text{)}), \end{array}$$ (3)

where *λ*(*τ*) represents the coefficient of spatial autocorrelation in the error term (*w*_*ij*_u_*i*_) and u_*i*_ the disturbance term or error term that exhibits spatial dependence or spatial impact.

3) Spatial quantile durbin model (SQDM)

This model takes into account the presence of spatial effects in both the explanatory variables and the response variable. In simpler terms, it considers that the variables being analyzed are not only influenced by their own values but also by the values of neighboring or nearby locations. The model can be presented as

$$\begin{array}{l} {\text{lnCASE}}_{i}=\rho (\tau )w_{ij}{\text{lnCASE}}_{i}+\beta_{1}\text{(}\tau {\text{)lnARRIVAL}}_{i}+\beta_{2}\text{(}\tau {\text{)lnAGE65}}_{i}+\beta_{3}\text{(}\tau {\text{)lnSTR}}_{i}+\beta_{4}\text{(}\tau {\text{)lnFULLVAC}}_{i}\\ \;\;\;\;\;\;\;\;\;\;\;\;\;\;\;\;\;\;\;\;+\beta_{5}\text{(}\tau {\text{)lnGDP}}_{i}+\beta_{6}\text{(}\tau {\text{)lnPOP}}_{i}+\beta_{7}\text{(}\tau \text{)}w_{i}{\text{lnTRADE}}_{i}+\theta_{1}\text{(}\tau \text{)}w_{i}{\text{lnARRIVAL}}_{i}+\theta_{2}\text{(}\tau \text{)}w_{i}{\text{lnAGE65}}_{i}\\ \;\;\;\;\;\;\;\;\;\;\;\;\;\;\;\;\;\;\;\;+\theta_{3}\text{(}\tau \text{)}w_{i}{\text{lnSTR}}_{i}+\theta_{4}\text{(}\tau \text{)}w_{i}{\text{lnFULLVAC}}_{i}+\theta_{5}\text{(}\tau \text{)}w_{i}{\text{lnGDP}}_{i}+\theta_{6}\text{(}\tau \text{)}w_{i}{\text{lnPOP}}_{i}+\theta_{7}\text{(}\tau \text{)}w_{i}{\text{lnTRADE}}_{i}+\varepsilon_{i},\;\;\;\;\varepsilon_{i}\sim N(0,\sigma^{2}\text{(}\tau \text{)}), \end{array}$$ (4)

where *θ*(*τ*) coefficient for the explanatory variables with spatial influence. In the estimation of SQDM, the parameters *β*_1_(*τ*) and *θ*(*τ*) cannot be directly interpreted due to the presence of bias in the estimation results [13]. Therefore, it is necessary to analyze and separate the effects of the explanatory variables into direct effects and indirect effects in order to determine their spatial impact. The direct effect would involve assessing how changes in international tourist arrivals in one country directly impact its own COVID-19 transmission rate. This provides insights into the immediate consequences of international tourism on a country’s public health situation. In contrast, the indirect effect explores how changes in international tourism in one country indirectly influence the COVID-19 transmission rates in neighboring countries. This indirect impact takes into account the spatial dependencies between countries, whereby the actions or conditions in one country may affect others through a network of spatial relationships. By considering both direct and indirect effects, this study can provide a comprehensive view of how international tourism potentially heightens COVID-19 transmission, not only within individual countries but also across borders, considering the interconnectedness of neighboring regions.

To obtain the direct and indirect effects, let Eq (4) be rewritten as the reduced form as follows:

$$\text{ln}{\text{CASE}}_{i}={\left(I-\rho W\right)}^{-1}[X_{i}\beta +WX_{i}\theta +\varepsilon_{i}]$$ (5)

where *I* is the *n* order identity matrix, *X*_*i*_ represents the set of the independent variables. According to LeSage and Pace[13], the partial derivatives of E(lnCASE_*i*_)with respect to each independent variable *k* is employed in this analysis. The general form is observed to be:

$$\begin{array}{ll} \left[\begin{array}{ccc} \frac{E\partial (\text{lnCASE})}{\partial x_{1k}} & \cdots & \frac{E\partial (\text{lnCASE})}{\partial x_{nk}} \end{array}\right] & ={\left(I-\rho W\right)}^{-1}\left[\begin{array}{ccc} \frac{E\partial (\text{ln}{\text{CASE}}_{1})}{\partial x_{1k}} & \cdots & \frac{E\partial (\text{ln}{\text{CASE}}_{1})}{\partial x_{nk}}\\ \vdots & \ddots & \vdots \\ \frac{E\partial (\text{ln}{\text{CASE}}_{n})}{\partial x_{1k}} & \cdots & \frac{E\partial (\text{ln}{\text{CASE}}_{n})}{\partial x_{nk}} \end{array}\right]\\ & ={\left(I-\rho W\right)}^{-1}\left[\begin{array}{cccc} \beta_{k} & w_{12}\theta_{k} & \cdots & w_{1n}\theta_{k}\\ w_{21}\theta_{k} & \beta_{k} & \cdots & w_{2n}\theta_{k}\\ \vdots & \vdots & \ddots & \vdots \\ w_{n1}\theta_{k} & w_{n2}\theta_{k} & \cdots & \beta_{k} \end{array}\right]. \end{array}$$ (6)

Then the direct effect can be obtained from the mean diagonal element multiplied by (*I* − *ρW*)^−1^, while the indirect effect is calculated as the mean row sum of off-diagonal elements times (*I* − *ρW*)^−1^.

The estimation will be conducted for quantiles ranging from the 10th to the 90th percentile. Each quantile has the following interpretations:

10th, 20th, 30th percentiles: The impact of tourism on infection rates in countries with low infection levels.
40th, 50th, 60th percentiles: The impact of tourism on infection rates in countries with moderate infection levels.
70th, 80th, 90th percentiles: The impact of tourism on infection rates in countries with high infection levels.

## 4. Results

The results of this study consist of the Moran I spatial autocorrelation test, followed by selecting the best-fit model using the BIC statistic. Subsequently, the study presents the estimated outcomes derived from the best-fit model, accompanied by an in-depth analysis of both direct and indirect factors that impact the COVID-19 infection rate across various quantiles. The details of each sub-section can be outlined as follows:

### 4.1 Moran I spatial autocorrelation test

In the initial part of this study, the researcher conducted a spatial autocorrelation test using the Moran I method. The study results are presented in Table 2, which shows that all variables have Moran I values greater than 0. This indicates significant positive spatial correlation, implying that there is a statistically significant and geographically influential relationship. Based on these test results, it can be concluded that changes in epidemic spread, population density, economic level, intensity of control measures, vaccination rates, tourism, and elderly population in one country will affect neighboring countries in the same direction. Therefore, the preliminary test confirms that spatial impact should be considered in this study.

**Table 2:** The Moran I test for each variable.

lnCASE	lnPOP	lnGDP	lnSTR	lnFULLVAC	lnARRIVAL	lnAGE65	lnTrade
0.2077*	0.4115***	0.3853***	0.0641*	0.3940***	0.3166***	0.6032***	0.2855***

Note

***, **, and * indicate the significance levels at 0.01, 0.05, and 0.10, respectively.

### 4.2 Comparison of spatial models’ performance

In this study, three spatial models are presented to obtain the best results at each quantile level. Therefore, it is necessary to compare the performance of each model. For this test, the Bayesian Information Criteria (BIC) will be considered, where the model with the lowest BIC value indicates the best model. Furthermore, to confirm the existence of spatial effects, this study also includes a comparison with the Quantile Regression model, which does not incorporate spatial effects. The results of the comparison are shown in Table 3, indicating that the spatial quantile durbin model (SQDM) performs the best at all quantile levels. This is evident from its lowest BIC value, suggesting that spatial influence is crucial in studying the impact of tourism on the spread.

**Table 3:** Test model identification.

BIC	10th	20th	30th	40^th^	50th	60th	70th	80th	90th
SQLM	330.45	320.89	304.01	290.10	280.37	265.01	273.19	254.63	239.71
SEQM	328.39	319.20	302.89	288.32	278.60	262.94	272.31	253.09	236.18
SDQM	**317.13**	**317.25**	**299.34**	**283.39**	**276.13**	**260.90**	**259.06**	**240.78**	**233.28**
Quantile Regression	334.31	319.00	302.34	292.32	283.91	279.14	276.91	278.65	285.05

**Note**: The bold number indicates the minimum BIC value.

Moreover, it confirms that the spatial effects influence both the spread and the number of tourists, along with other control variables. Therefore, the SDQM model will be used to analyze the spatial impact of international tourists on the spread in the subsequent analysis.

### 4.3 Estimation results of SQDM

Based on the findings presented in the previous section, it is evident that the SDQM proves to be the most appropriate model for the data utilized in this study. Furthermore, it is essential to apply the Likelihood ratio (LR) test to validate that the spatial correlation pattern is adequately captured by the SDQM. The results of the LR test, displayed in the final two rows of Table 4, indicate significant outcomes at the 0.01 significance level. Additionally, it is evident that the SDQM cannot be simplified or reduced to either the SQLM or SEQM.

**Table 4:** Estimated results of the SDQM.

		10th	20th	30th	40th	50th	60th	70th	80th	90th
**direct effect**	(Intercept)	-1.168	-3.045***	-17.330*	-24.088***	20.011***	-21.809***	-22.714***	-19.759**	-19.313***
		(1.869)	(0.501)	(9.679)	(8.749)	(5.803)	(8.042)	(7.523)	(8.415)	(7.429)
	lnARRIVAL	0.124	0.050	0.039	0.012	0.126	0.109	0.436*	0.216**	0.117**
		(0.272)	(0.255)	(0.204)	(0.198)	(0.107)	(0.180)	(0.261)	(0.108)	(0.059)
	lnGDP	1.089*	0.636**	0.491**	0.749**	0.396*	0.826*	0.549 **	0.456	0.374
		(0.541)	(0.305)	(0.205)	(0.316)	(0.160)	(0.485)	(0.255)	(0.536)	(0.441)
	lnSTR	-0.285	-0.102	-0.069	-0.294	-0.010	-0.545	-0.779**	-0.560	-0.438
		(0.754)	(0.708)	(0.553)	(0.516)	(0.040)	(0.459)	(0.352)	(0.553)	(0.509)
	lnFULLVAC	-0.268***	-0.198***	-0.311	0.607	-0.818*	0.989	0.001	-0.858***	-0.484*
		(0.016)	(0.078)	(0.802)	(0.840)	(0.360)	(0.858)	(0.023)	(0.401)	(0.789)
	lnPOP	0.483	0.306**	0.264**	0.087***	0.313*	0.197	0.893*	0.050***	0.081***
		(0.302)	(0.152)	(0.106)	(0.021)	(0.128)	(0.212)	(0.504)	(0.023)	(0.008)
	lnAGE65	0.758*	1.924**	1.737**	1.200*	1.236***	1.015*	0.263*	0.633***	0.799*
		(0.301)	(0.860)	(0.736)	(0.713)	(0.380)	(0.583)	(0.155)	(0.175)	(0.419)
	lnTRADE	0.149	0.088	0.281**	0.442**	0.714	0.489	0.559*	0.329	0.821*
		(0.662)	(0.659)	(0.115)	(0.211)	(0.475)	(0.463)	(0.342)	(0.500)	(0.443)
**Indirect effect**	WlnARRIVAL	0.910*	0.149	0.023	-0.110	0.076	0.109	-0.047***	-0.050***	-0.047***
		(0.512)	(0.324)	(0.207)	(0.194)	(0.137)	(0.171)	(0.017)	(0.019)	(0.016)
	WlnGDP	-0.992	-0.308	0.208	0.473	0.382	0.230	0.604	0.416*	0.093
		(1.060)	(1.327)	(0.624)	(0.618)	(0.329)	(0.686)	(0.500)	(0.256)	(0.455)
	WlnSTR	0.704	-0.646	-0.278	-0.096*	-0.608**	-0.281***	-0.316	-0.268	-0.102
		(1.076)	(0.992)	(0.897)	(0.051)	(0.108)	(0.078)	(0.851)	(0.897)	(0.775)
	WlnFULLVAC	-0.177***	-0.262***	-0.180***	1.143	-0.779	-0.233	0.809	0.930	1.379
		(0.028)	(0.048)	(0.061)	(1.171)	(0.344)	(1.507)	(1.615)	(1.739)	(1.354)
	WlnPOP	-0.226	0.004	-0.033	-0.306	0.125	0.111	-0.284	-0.124	-0.081
		(0.473)	(0.005)	(0.355)	(0.294)	(0.205)	(0.310)	(0.309)	(0.335)	(0.281)
	WlnAGE65	0.132	-0.167	0.106	0.228	-0.188	0.027	-0.025	0.095	0.028
		(1.040)	(0.990)	(0.668)	(0.850)	(0.450)	(0.988)	(0.972)	(1.056)	(0.792)
	WlnTRADE	-0.815	-0.977*	-0.576	-0.293	-0.554	-0.143	-0.527	-0.649*	-0.797*
		(0.980)	(0.457)	(0.783)	(0.673)	(0.483)	(0.642)	(0.625)	(0.361)	(0.410)
	*ρ*	0.490*	0.370**	-0.133***	-0.005	0.008*	-0.051**	-0.155***	-0.037***	0.037**
		(0.216)	(0.175)	(0.021)	(0.004)	(0.04)	(0.018)	(0.021)	(0.013)	(0.012)
LR-spatial lag		72.82***	47.95***	48.98***	51.02***	55.55***	66.42***	48.44***	58.16***	50.74***
LR-spatial error		53.16***	37.40***	39.00***	40.38***	44.92***	55.49***	38.70***	47.76***	38.35***

Note:

***, **, and * indicate significance at the 0.01, 0.05, and 0.10 significance levels, respectively.

The estimated results from the SDQM model are presented in Table 4, demonstrating the impacts of tourism and other relevant factors on the spread at different quantile levels. The study’s findings can be divided into two parts: direct impacts and indirect impacts. The direct impacts refer to the effects of various factors that directly influence the country itself. On the other hand, the indirect impacts represent the influences of different factors that spill over to neighboring countries.

Table 4 reveals compelling findings regarding the direct impacts. Firstly, it highlights that the influx of international tourists (ARRIVAL) significantly influences the COVID-19 infection rate within countries experiencing high infection rates, specifically in the 70th-90th quantile range. This emphasizes the role of tourism as a contributing factor to the spread of the virus within heavily affected regions. Moreover, when considering other pertinent variables, we find that the population (POP) variable exhibits a noteworthy impact on the infection rate in countries with a relatively lower to moderate infection rate, within the 20th-40th quantile range, as well as in countries with high infection rates, falling within the 70th-90th quantile range. This highlights the population size as a crucial determinant of the spread in both moderately affected and highly impacted regions.

Furthermore, the analysis indicates that the GDP variable influences the infection rate in countries with a lower to moderate infection rate, encompassing the 10th-70th quantile range. This suggests that economic factors play a role in shaping the spread of COVID-19 in regions with varying infection levels. The FULLVAC variable demonstrates a substantial impact on the COVID-19 infection rate in countries across different quantile ranges. Specifically, it affects the infection rate in countries with low infection rates (10th-20th quantile range), moderate infection rates (50th quantile range), and high infection rates (80th-90th quantile range). This emphasizes the importance of vaccination coverage in mitigating the transmission of the virus across diverse settings. Regarding the AGE65 variable, it is observed to have a significant influence on the infection rate in countries with lower to moderate infection rates, within the 10th-60th quantile range. This highlights the vulnerability of elderly populations to COVID-19 and underscores the importance of considering age-related factors in pandemic management strategies. Importantly, the analysis reveals that the STR variable does not exhibit a statistically significant impact on the COVID-19 infection rate. This suggests that the stringency of containment measures alone may not be a significant determinant in the spread of the virus. In term of TRADE, a significant impact of trade openness on the COVID-19 infection rate is observed at the 30th, 40th, 70th, and 90th quantiles.

In addition to the direct impacts mentioned earlier, Table 4 also provides insights into the indirect impacts. It reveals that the variable "WARRIVAL" has an influence on the spread of the infection to neighboring countries, particularly in countries with high infection rates at the 70th-90th quantile level. Among other related variables, "WGDP" has a significant impact on neighboring countries, especially in countries with high infection rates at the 80th quantile level. The variable "WSTR" affects neighboring countries in countries with moderate infection rates at the 40th-60th quantile level. Lastly, the variable "WFULLVAC" impacts neighboring countries in countries with low infection rates at the 10th-30th quantile level.

Based on the above results, only the significant direct and indirect impacts are presented. However, it is not yet possible to interpret the estimated coefficients obtained from the estimation process. This is because the estimated results may still be biased, and the interpretation does not have economic significance. Therefore, it is necessary to transform the estimated results using the spatial-weighted derivative method, which compares the expected value of the weighted spread of the infection with the explanatory variables within the same and different areas[13]). The estimated results of both direct and indirect impacts will be presented in the subsequent section.

### 4.4 Robustness checks

To validate the findings of this study, it is essential to account for the endogeneity problem. This concern arises due to the potential bidirectional causation between tourism arrivals and COVID-19 transmission, as well as the possibility that omitted variables can contribute to the endogeneity problem in the economic model [27]. Following the approach recommended by Lee and Yu [39], the Two-Stage Least Squares (2SLS) technique is employed to mitigate the endogeneity problem when estimating the SDQM. In this methodology, the real effective exchange rate (REER) is considered a suitable instrumental variable. The choice is based on the fact that the REER is not only used as a viable proxy for tourism arrivals but is also intricately linked to personal travel patterns, which are more relevant to tourism than to the transmission of COVID-19 [40, 41]. In the first stage, tourism arrivals are estimated using the instrumental variable as a regressor. Subsequently, the expected tourism arrivals are utilized as a regressor in the SDQM equation. It is crucial to note that the REER data utilized in our analysis were sourced from the reputable World Bank database.

To ensure the robustness of the real effective exchange rate (REER) as the chosen instrumental variable, the F-statistics test was performed during the initial stage of estimation. When the F-statistic exceeds 10, as per the "rule of thumb" critical value recommended by Wang and Zivot [42], the null hypothesis of a weak instrument is rejected, thereby indicating the strength of our chosen instrument. Additionally, the Durbin-Wu-Hausman test, as outlined by Hausman [43], is employed to assess whether the one-step estimation of the SDQM is both efficient and consistent. The rejection of the null hypothesis in this test implies that the one-step estimation of the SDQM is inconsistent, suggesting the presence of endogeneity.

Table 5 presents the results for the SDQM model employing the instrumental variable (REER), along with the results of the endogeneity tests in the final two rows. It is observed that the statistical values of the Durbin-Wu-Hausman test are all insignificant, while the F-statistics significantly exceed the critical value of the test (i.e., 10). This implication establishes the robustness of the chosen instrumental variable and indicates the absence of an endogeneity issue in our empirical model. Furthermore, it is noted that the coefficients of tourism arrival remain consistent with the results presented in Table 4. The results of the robustness tests, as displayed in Table 5, reveal that the positive effect of tourism arrival on COVID-19 transmission remained significant at higher quantile levels, thus affirming the robustness of the benchmark regression results presented in Table 4.

**Table 5:** Estimated results of the SDQM with instrument variable (REER).

		10th	20th	30th	40th	50th	60th	70th	80th	90th
**Direct effect**	(Intercept)	2.194*	-3.0821***	-15.204*	-15.895***	13.509*	-10.550**	-15.103***	-11.903***	-19.313***
		(1.301)	(1.468)	(8.164)	(5.990)	(8.109)	(5.293)	(6.113)	(4.709)	(5.031)
	lnARRIVAL	0.193	0.030	0.190	0.053	0.410	0.693	0.441*	0.193*	0.206*
		(0.378)	(0.259)	(0.204)	(0.201)	(0.309)	(1.340)	(0.289)	(0.116)	(0.121)
	Control variables	controlled								
**Indirect effect**	WlnARRIVAL	-0.598*	0.149	0.023	-0.344	0.209	0.301	-0.110***	-0.139***	-0.072*
		(0.330)	(0.524)	(0.407)	(0.301)	(0.278)	(0.209)	(0.029)	(0.061)	(0.041)
	WlnControl variables	controlled								
	*ρ*	1.091***	0.420*	-0.133***	-0.015	0.089**	-0.051**	-0.155***	-0.157***	0.041**
		(0.390)	(0.275)	(0.056)	(0.014)	(0.040)	(0.025)	(0.036)	(0.043)	(0.022)
	F statistic test	23.948	19.993	10.982	21.029	23.092	16.083	19.987	20.873	26.093
	Durbin-Wu-Hausman test	2.813	1.923	1.002	2.013	1.628	2.083	0.972	1.119	1.092

Furthermore, a comparison between the full model, presented in Table 4, and the model that excludes control variables, depicted in Table 6, allows for an assessment of the impact of controlling for potential endogeneity and omitted variable bias. Notably, in the absence of control variables, the results from the model in Table 6 reveal consistent and robust relationships between the variables across all quantile levels. The sizes, signs, and levels of significance of the coefficients remain largely unchanged, underscoring the stability and validity of the findings. This comparative analysis suggests that accounting for potential endogeneity and omitted variable bias through control variables does not substantially alter the observed relationships between the variables. Consequently, it can be inferred that the direct relationship between the variables of interest remains robust and unaffected by the inclusion or exclusion of control variables, further enhancing the reliability of this study.

**Table 6:** Estimated results of the SDQM without control variables.

		10th	20th	30th	40th	50th	60th	70th	80th	90th
**Direct effect**	(Intercept)	4.965**	-2.928***	-12.116*	-15.012***	12.357***	-10.893***	-14.309***	-12.093***	-19.313***
		(0.522)	(0.338)	(7.913)	(4.983)	(4.835)	(4.109)	(5.309)	(4.072)	(4.023)
	lnARRIVAL	0.050	0.045	0.045	0.067	0.089	0.578	0.339*	0.120**	0.303*
		(0.255)	(0.243)	(0.107)	(0.123)	(0.097)	(0.988)	(0.022)	(0.062)	(0.102)
	Control variables	Uncontrolled								
**Indirect effect**	WlnARRIVAL	-0.556*	0.208	0.067	-0.210	0.132	0.273	-0.065***	-0.093***	-0.041*
		(0.317)	(0.462)	(0.378)	(0.237)	(0.206)	(0.187)	(0.020)	(0.035)	(0.033)
	WlnControl variables	Uncontrolled								
	*ρ*	1.223***	0.609***	-0.103**	0.033***	0.089***	0.039**	-0.104***	-0.103***	0.030**
		(0.326)	(0.241)	(0.032)	(0.011)	(0.020)	(0.020)	(0.029)	(0.036)	(0.015)

### 4.5 Factors affecting direct and indirect impacts on the COVID-19 infection rate

Table 7 presents the factors directly impacting the COVID-19 infection rate. Specifically, the number of international tourists (ARRIVAL) is found to be statistically significant in relation to the COVID-19 infection rate, particularly in countries with high infection rates at the 70th-90th quantile level. The estimation results indicate that a 1%increase in ARRIVAL is associated with an increase in the infection rate per population by 0.117–0.436%. This study provides insights into the concept that tourism involves the movement of people between different locations, and tourists can act as carriers of diseases. Consequently, they have the potential to spread the disease to the local population at their destination.

**Table 7:** Direct effects.

DIRECT	10th	20th	30th	40th	50th	60th	70th	80th	90th
ARRIVAL	0.183	0.077	0.045	0.019	0.145	0.093	0.502*	0. 251**	0.154*
GDP	1.384*	0.935**	0.502*	0.832**	0.475*	0.899*	0.603 **	0.040	0.294
STR	-0.274	-0.135	-0.008	-0.108	-0.001	-0.661	-0.903**	-0.294	-0.504
FULLVAC	-0.309***	-0.330***	-0.356	0.811	-0.904*	0.673	0.001	-0.923***	-0.741*
POP	0.501	0.337**	0.289*	0.209***	0.381*	0.209	1.034*	0.119**	0.105***
AGE65	0.812*	2.149**	1.800***	1.283*	1.360***	1.137*	0.638*	0.891***	0.903**
TRADE	0.101	0.007	0.306***	0.515**	0.805	0.506	0.577*	0.361	0.883*

Note:

***, **, and * indicate significance at the 0.01, 0.05, and 0.10 significance levels, respectively.

Regarding other related factors, population density (POP) has been found to have an impact on the COVID-19 infection rate in countries with low to moderate infection rates (quantiles 20th-50th) and countries with high infection rates (quantiles 70th-90th). If the population increases by 1%, it leads to an increase in the infection rate per population by 0.209–0.381% in the former group and by 0.105–1.034% in the latter group. These results are in line with the anticipated outcomes and the natural transmission of infectious diseases. In other words, higher population density contributes to population congestion, which facilitates the spread of the virus. Several studies from the past to the present support these findings. For example, Fang et al. [19] found that population density was a key factor influencing the SARS infection in Beijing. The latest study by Ehlert [30] also demonstrated the positive association between population density and COVID-19 infection. Therefore, it can be concluded that population density plays a role in the COVID-19 infection rate, covering almost all groups of countries.

Regarding the variable of Gross Domestic Product (GDP), it has an impact on the COVID-19 infection rate in countries with low to moderate infection rates (quantiles 10th-30th) and countries with moderate to high infection rates (quantiles 40th-70th). If GDP increases by 1%, it leads to an increase in the infection rate per population by 0.502–1.384% in the former group and by 0.475–0.899% in the latter group. These findings align with the research conducted by You, Wu, and Guo [44] and Ehlert [30], which stated that economic activities such as employment, retail sales per unit area, and industrial value have a positive impact on infection and mortality rates from contagious diseases. This is because the level of economic activities in a country often involves the gathering of individuals, which can lead to easy transmission of infectious diseases.

The variable of full vaccination rate per population (FULLVAC) also has an impact on the COVID-19 infection rate in countries with low, moderate, and high infection rates. In the quantiles 10th-20th, an increase of 1% in FULLVAC results in a percentage decrease of 0.309–0.330 in the infection rate per population. At the 50th quantile, a 1% increase in FULLVAC leads to a percentage decrease of 0.904 in the infection rate per population. In the quantiles 80th-90th, a 1% increase in FULLVAC causes a percentage decrease of 0.741–0.923 in the infection rate per population. These findings are consistent with the expected outcomes. It is worth noting that in the group of countries with a moderate infection rate, an increase in vaccination rate leads to a greater reduction in the infection rate per population compared to other country groups.

In the case of the variable representing the proportion of the population aged 65 and above (AGE65), it is found to have a positive impact on the COVID-19 infection rate in countries with low to moderate infection rates. In the quantiles 10th-30th, a 1% increase in AGE65 leads to a percentage increase of 0.812–2.149 in the infection rate per population. At the 40th-60th quantiles, a 1% increase in AGE65 results in a percentage increase of 1.137–1.360 in the infection rate per population. Likewise, at the 70th-90th quantiles, a 1% increase in AGE65 results in a percentage increase of 0.638–0.903 in the infection rate per population. This could be because the elderly population is more susceptible to contracting infectious diseases due to their lower immune defenses compared to other age groups [45]. The finding that the stringency index of control measures (STR) had a statistically significant impact on the COVID-19 infection rate at multiple quantiles signifies its versatile influence. Notably, this impact is observed at the 10th, 20th, 50th, 80th, and 90th quantiles, indicating that the stringency of control measures effectively influence infection rates across a wide range of conditions. This means that stricter control measures are associated with significant changes in infection rates not only when rates are low but also when they are moderate or high. Furthermore, trade openness is found to positively influence the infection rate at the 30th, 40th, and 70th quantiles.

Indirect effects analysis considers the impact between the different regions or countries, particularly those that share borders or are in close proximity. This is shown in Table 8, where it is found that the number of international tourists (ARRIVAL) affects the spread of the infection to neighboring countries, especially in countries with high infection rates at the 70^th^ and 90^th^ percentiles. This is consistent with the direct effects observed. However, it is interesting to note that the impact of the number of international tourists from one country can have a negative effect on neighboring countries. In other words, an increase of 1% ARRIVAL can lead to a decrease in the infection rate per population in neighboring countries by 0.063–0.127%.

**Table 8:** Indirect effects.

INDIRECT	10^th^	20^th^	30^th^	40^th^	50^th^	60^th^	70^th^	80^th^	90^th^
ARRIVAL	0.998*	0.190	0.012	-0.103	0.054	0.093	-0.104***	-0.127***	-0.063***
GDP	-0.832	-0.332	0.192	0.532	0.261	0.290	0.677	0.532*	0.081
STR	0.798	-0.712	-0.301	-0.112*	-0.710***	-0.324*	-0.351	-0.291	-0.032
FULLVAC	-0.188***	-0.268***	-0.185***	1.143	-0.799	-0.284	0.823	0.967	1.441
POP	-0.227	0.001	-0.003	-0.213	0.139	0.081	-0.301	-0.143	-0.042
AGE65	0.146	-0.171	0.130	0.249	-0.301	0.042	-0.031	0.105	0.071
TRADE	-0.904	-1.384*	-0.570	-0.224	-0.554	-0.151	-0.530	-0.657*	-0.801*

Note:

***, **, and * indicate significance at the 0.01, 0.05, and 0.10 significance levels, respectively.

For other control variables, it is found that Gross Domestic Product (GDP) per capita has an impact on the spread of the infection to neighboring countries in the group of countries with high infection rates at the 80th percentile. Specifically, if GDP increases by 1%, it leads to an increase in the infection rate per population in neighboring countries by 0.532%. However, this effect is observed only in the group of countries with high infection rates. It can be said that when a country increases its GDP per capita, it attracts foreign labor from neighboring countries. However, countries with such characteristics are those with high infection rates within the country (at the 80th percentile). Therefore, the incoming foreign workforce in these countries is at risk of contracting the infection. When these foreign workers travel back to their home countries, they may carry the disease with them.

In addition to that, the Stringency Index (STR) is found to have an impact on the spread of the infection to neighboring countries in the group of countries with moderate infection rates at the 40th-60th percentile. Specifically, if the STR increases by 1%, it leads to a decrease in the infection rate per population in neighboring countries by 0.112–0.710%. This indicates that the intensity of controlling the spread of the infection in one country has a positive effect on neighboring countries. This finding is consistent with the study by Gordon, Grafton, and Steinshamn [46], which explained that stringent control measures in one country make neighboring countries more vigilant and reduce cross-border activities, resulting in a significant reduction in the spread of the infection.

Lastly, the full vaccination rate per population (FULLVAC) has an influence on the spread of the infection to neighboring countries in the group of countries with low infection rates at the 10th-30th percentile. Specifically, if the FULLVAC increases by 1%, it leads to a decrease in the infection rate per population in neighboring countries by 0.118–0.268%. This finding is in line with the control and prevention measures (STR) and demonstrates that increasing the vaccination rate significantly reduces the spread of the infection to neighboring countries.

**Fig 2:**
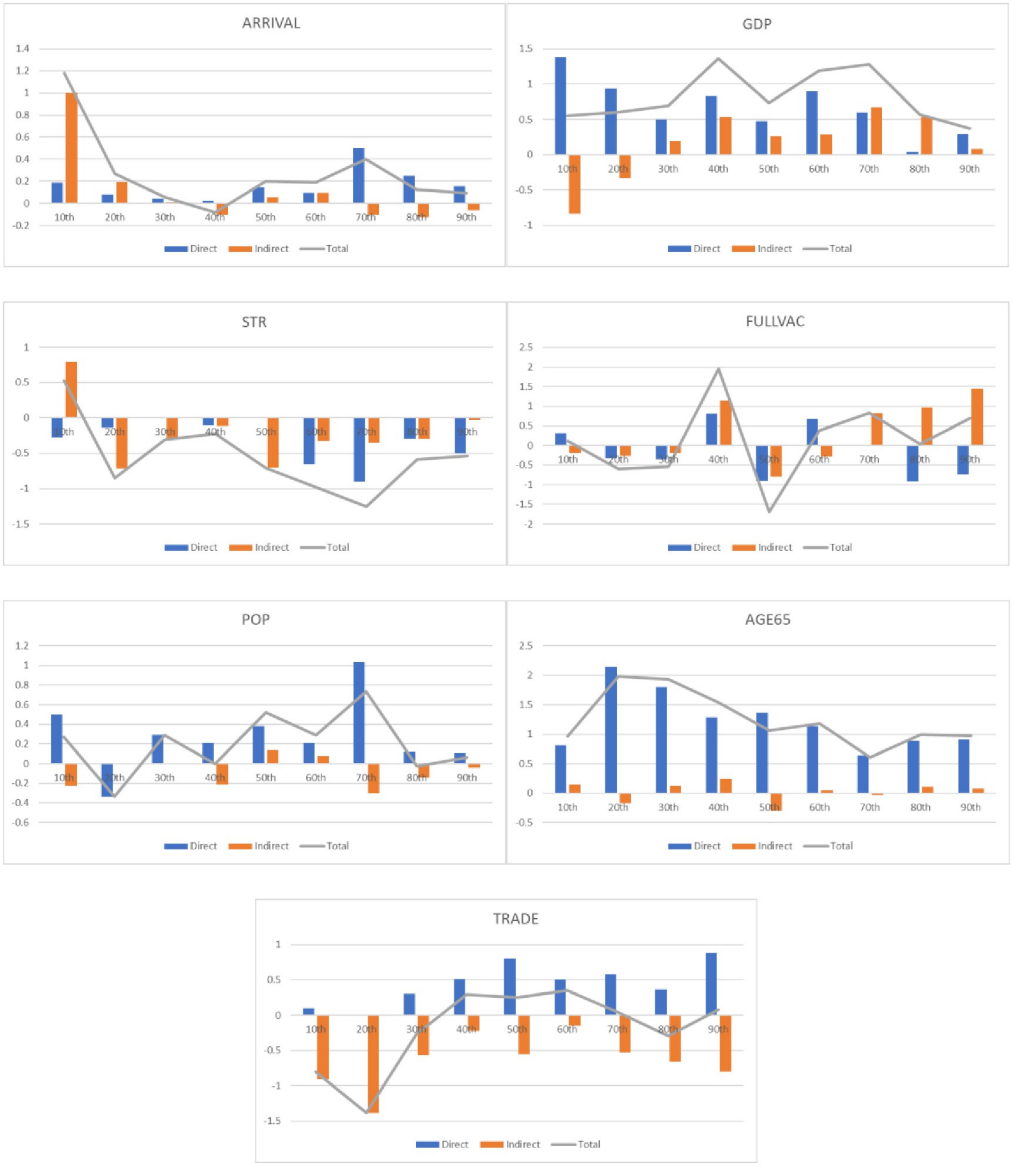
Impact of different variables on the rate of COVID-19 infection from the 10th to the 90th quantiles. **Note**: Blue bars represent the direct impact on the rate of COVID-19 infection. Orange bars represent the indirect impact on the rate of COVID-19 infection. Gray lines represent the overall impact on the rate of COVID-19 infection.

To demonstrate the clear findings of this study, the overall analysis results are illustrated in Fig 2, which includes both direct and indirect effects, as well as the overall impact. The results for each variable studied are as follows

## 5. Discussion

During periods when the disease situation begins to stabilize, each country faces the critical question of whether they are prepared to reopen their borders or not. Assessing a nation’s preparedness for border reopening necessitates the evaluation of several key factors. Among these factors, the tourist arrival stands out as a core variable, offering insights into a country’s reliance on tourism. Additionally, economic indicators, vaccination rates, stringency indices reflecting the strictness of epidemic control measures, the proportion of the elderly population, and the degree of trade openness serve as crucial control variables in this assessment.

In this study, we examine the effects of tourism arrivals on COVID-19 spread, breaking down these impacts into direct and indirect effects. The results reveal that the number of international tourists has both direct and indirect effects on COVID-19 infection rates, particularly in countries experiencing high infection rates falling within the 70th to 90th quantile level. This indicates that higher tourism arrivals can contribute to an elevated spread of COVID-19 within local countries, especially those with a high existing infection rate, as well as in their neighboring countries.

The findings of this study align with previous research conducted by Findlater and Bogoch [5], which highlights the effective spread of infectious diseases through travel. Factors such as increased human mobility through trade, migration, and tourism contribute to the spread of diseases. Moreover, the study by Tantrakarnapa, Bhopdhornangkul, and Nakhaapakorn [7] supported the notion that the number of tourists, along with their activities, significantly correlates with the number of COVID-19 cases and patients. It is important to recognize that airports and important tourist destinations serve as critical hubs for disease transmission. Thus, increased tourism has a positive impact on the spread of the pandemic. However, this study has uncovered an intriguing pattern: the significant impact of hosting a large number of foreign tourists on infection spread is primarily observed in countries with high infection rates, particularly those situated within the 70th-90th quantile level. These results align with the findings of Bickley et al. [18], who supported the notion that the degree of COVID-19 spread can vary depending on healthcare capacity and globalization. Higher levels of both factors could make countries more susceptible to infectious disease outbreaks. Therefore, in countries where public health factors and control measures have not effectively contained the virus, welcoming more tourists can lead to an increase in the infection rate among the population. Conversely, in countries that have successfully controlled the infection rate to a low level, it is possible to open up to tourism while maintaining stringent control measures. This approach aims to ensure that the infection rate remains at a similar level as before, preventing surges in cases even as tourists arrive.

It is crucial to recognize the essential role of control and protection measures following the reopening of borders, as emphasized by Yu et al. [47]. In countries already contending with high infection rates, the absence of these additional safeguards could result in a significant and rapid increase in COVID-19 infections. This, in turn, would lead to a higher number of cases, increased hospitalizations, and an increase in the number of deaths. Therefore, if a country reopens its borders without the implementation of extra protective measures to address this issue, the resurgence of tourism could worsen the severity of the ongoing COVID-19 pandemic within that country. This highlights the critical importance of adopting a comprehensive and well-balanced strategy that considers both economic factors and public health imperatives when deciding to reopen borders [48].

Regarding the indirect effects, the influx of tourists into one country also has an impact on its neighboring countries, particularly those with high infection rates. This phenomenon can be attributed to the implementation of long-term travel restrictions by countries during severe outbreaks. Consequently, when a country successfully manages its outbreak situation or faces economic pressures, it may choose to reopen its borders without taking into account the measures implemented by neighboring countries. In such cases, countries may employ various strategies to attract international tourists [49]. It can be observed that this practice of focusing on a single destination can lead to localized outbreaks within that country while reducing the number of infections in neighboring countries by comparison.

As a result, this study offers a partial answer to the research problem, indicating that countries with moderate or low infection rates have the potential to cautiously reopen their borders, taking into account the potential risk of low-level transmission. However, it may not be advisable for countries with high infection rates to open up at this time, as tourism exhibits a significant impact on virus spread within the country. These findings highlight the importance of considering the specific context and infection situation of each country when making decisions regarding the resumption of tourism activities. It emphasizes the need for tailored strategies that account for the effectiveness of public health measures and control mechanisms in managing infection rates in relation to tourism. Future research can delve deeper into exploring the optimal timing and conditions for reopening tourism in different infection rate contexts, contributing to the development of evidence-based policies in the face of the ongoing pandemic.

## 6. Limitations and recommendations for future research

This study has some limitations that should be acknowledged. Firstly, it primarily focuses on the short-term effects of the pandemic, explicitly considering the year 2022. Consequently, it cannot provide insights into the medium and long-term implications of the pandemic. Future research is suggested to consider longer-span data, extending over at least three years, to comprehensively assess the lasting impacts of COVID-19 on the tourism industry. Additionally, employing panel spatial models may better accommodate the panel data. Another limitation is the relatively limited dataset, which includes data from only 83 countries. To enhance the accuracy of classifications based on infection rates, it is recommended that future research includes data from additional countries. This expanded dataset would facilitate a more precise analysis and a broader understanding of the factors influencing the spread of the virus. Moreover, including data from a more significant number of countries can enable the examination of the heterogeneous impacts of COVID-19, which may vary across regions. The use of panel spatial quantile models is also recommended in this regard. Thirdly, this study uses countries as the statistical unit, which may overlook territorial variations. While finer territorial units, such as regions or provinces, could offer more detailed insights, obtaining comprehensive provincial-level data for all countries in our sample was not feasible. This limitation restricts our ability to capture localized patterns and assess the impact of tourist arrivals on COVID-19 cases at a more granular level. Future research could explore regional variations where data is available to understand the relationship better.

Finally, the study’s constrained timeframe and limited data availability for variables such as convalescence, the number of immune individuals, and COVID-19 testing present significant challenges. This study acknowledges the pivotal roles of these factors in influencing the spread of COVID-19, as suggested in Eichenberger et al. [50]. Consequently, if future research can access more extensive data from a broader range of countries, it is recommended to consider including these variables in the analysis. Future research could explore the impact of new COVID-19 variants and strains on international tourism and virus transmission. T

## 7. Conclusion and policy recommendations

The global spread of COVID-19 since 2020 has led many countries to implement various measures to control the situation, particularly the restriction of human mobility to limit the transmission of the virus from person to person. This has resulted in significant damage to the tourism industry over the past 2–3 years. Therefore, as the pandemic begins to recede, many countries are making efforts to reopen their borders to stimulate the economy and revive their tourism industry. However, reopening the countries also comes with the risk of another wave of infections in many parts of the world, raising the question of whether we are ready to reopen our countries.

This question has arisen repeatedly, and a conclusive answer regarding the readiness to reopen our countries remains inconclusive. To tackle this concern, data pertaining to tourism and the virus’s spread in 2022 were gathered from 83 countries across the globe. This timeframe aligns with the period during which numerous countries initiated the process of reopening their borders.

The tool used in this study is the spatial quantile model, which is capable of analyzing the spatial impact of tourism on the spread of the virus at different quantile levels. The research divided countries into three categories: low infection rate (10th-30th quantiles), moderate infection rate (40th-60th quantiles), and high infection rate (70th-90th quantiles). This allowed for a more comprehensive and detailed comparison of the impacts. Additionally, considering the spatial dimension enabled the explanation of both direct and indirect impacts of tourists on the country itself and neighboring countries.

Findings and policy implications can be summarized as follows:

1. For countries with low infection rates, this study found that the number of international tourists did not have a significant impact on the country’s infection rate. Instead, factors such as income per capita, population density, vaccination coverage rate, and the proportion of the elderly population had significant effects on the infection rate. These internal factors played a crucial role in determining the spread of the virus within the country. The government should prioritize economic factors and GDP per capita, while also taking measures to reduce population density and monitor vaccination coverage and care for the elderly. Failure to manage these aspects properly can have negative consequences for the country and its neighbors. Countries in this group can consider reopening their borders with limitations on the number of tourists while closely monitoring relevant variables for sustainable economic recovery.
2. For countries with moderate infection rates, the number of international tourists did not significantly impact the spread of the virus. Instead, variables such as GDP per capita, population density, complete vaccination coverage, and the population aged 65 and above were more influential. Countries in this group may consider reopening their borders but should prioritize care for the elderly, reduce population gatherings, support complete vaccination coverage, and enforce preventive measures to minimize domestic transmission. The stringency of preventive measures also has a significant impact on neighboring countries, so it should be given attention to minimize negative consequences.
3. In the case of high infection rates, our analysis revealed that tourist arrivals had a significant direct and indirect impact on the spread of the virus in domestic and neighboring countries. This suggests that countries with high infection rates should delay reopening their borders to tourists to prevent further outbreaks. The number of international tourists not only affects domestic transmission but can also impact neighboring countries, potentially leading to higher infection rates. This study suggests that once the number of infections decreases, reopening borders can be considered to facilitate economic recovery. Moreover, instead of focusing on international reopening initiatives, nations should prioritize sustaining their economy through domestic tourism. Consequently, nations should shift their focus towards bolstering their economy by promoting domestic tourism
4. Considering the control variables, the results showed that the proportion of elderly individuals aged 65 and above had a significant impact on the infection rate in all quantile levels. Governments should prioritize this vulnerable group, accelerate vaccine distribution, and provide accurate information to combat vaccine misinformation. Expediting vaccination efforts for the general population is also crucial.
5. In terms of indirect effects, epidemic control measures and vaccination rates were found to impact neighboring countries. Effective control measures and increasing vaccination rates can lead to decreased infection rates in neighboring countries. Therefore, the source or main countries should promote public health measures, contribute to vaccine donation, and provide assistance to neighboring countries. Comprehensive and vigilant public health measures should be maintained within the country. Additionally, the number of incoming tourists from the source country was found to significantly affect the infection rate of neighboring countries. Neighboring countries become more concerned when a high-infection-rate source country opens its borders to tourists, prompting them to strengthen public health policies and measures. Source countries with a motivation to reopen for tourism also strive to keep their infection rates low. Promote public health measures and contribute to vaccine donations for neighboring countries.

In conclusion, our study contributes valuable guidance for policymakers and researchers alike in addressing the intricate challenges posed by COVID-19 and international tourism. By tailoring strategies to infection rates and considering future research avenues, our objective is to provide insights that can guide decision-making and fortify the resilience of the global tourism industry in light of the ongoing uncertainties.

## Supporting information

S1 Appendix (DOCX)

S1 Data (XLSX)
