# Supplementary material for: Does the resumption of international tourism heighten COVID-19 transmission?
Source: PLoS One. 2024 Feb 7;19(2):e0295249. doi: 10.1371/journal.pone.0295249 (PMC10849229; doi:10.1371/journal.pone.0295249)
Supplement: S1 Appendix — (DOCX) [file pone.0295249.s001.docx]

**Appendix**

**Table A1. List of countries**

| Africa | Asia | Europe | North America | Oceania | South America |
| --- | --- | --- | --- | --- | --- |
| Eswatini | Bahrain | Albania | Bahamas | Australia | Argentina |
| Mauritius | Cambodia | Austria | Barbados | New Zealand | Bolivia |
| Morocco | Georgia | Belgium | Belize |  | Colombia |
| Namibia | Hong Kong | Bosnia and Herzegovina | Bermuda |  | Ecuador |
| Seychelles | India | Bulgaria | Canada |  | Paraguay |
| South Africa | Indonesia | Croatia | Costa Rica | | Peru |
| Tanzania | Israel | Cyprus | Dominican Republic | | Uruguay |
| Tunisia | Japan | Estonia | El Salvador | |  |
|  | Jordan | Finland | Guatemala | |  |
|  | Kazakhstan | France | Honduras |  |  |
|  | Macao | Germany | Mexico |  |  |
|  | Malaysia | Greece | Panama |  |  |
|  | Nepal | Hungary | United States | |  |
|  | Philippines | Italy |  |  |  |
|  | Qatar | Kosovo |  |  |  |
|  | Saudi Arabia | Latvia |  |  |  |
|  | Singapore | Luxembourg | |  |  |
|  | South Korea | Malta |  |  |  |
|  | Sri Lanka | Netherlands | |  |  |
|  | Taiwan | Poland |  |  |  |
|  | Thailand | Portugal |  |  |  |
|  | Turkey | Romania |  |  |  |
|  | Uzbekistan | Russian Federation | |  |  |
|  | Vietnam | Serbia |  |  |  |
|  |  | Slovenia |  |  |  |
|  |  | Spain |  |  |  |
|  |  | Switzerland | |  |  |
|  |  | Ukraine |  |  |  |
|  |  | United Kingdom | |  |  |
